# Insights into the Formation of Intermolecular Complexes of Fluorescent Probe 10-*N*-Nonyl Acridine Orange with Cardiolipin and Phosphatidylglycerol in Bacterial Plasma Membrane by Molecular Modeling

**DOI:** 10.3390/molecules28041929

**Published:** 2023-02-17

**Authors:** Ekaterina Kholina, Ilya Kovalenko, Andrew Rubin, Marina Strakhovskaya

**Affiliations:** Faculty of Biology, Lomonosov Moscow State University, 119234 Moscow, Russia

**Keywords:** 10-*N*-nonyl acridine orange (NAO), lipid probe, model bacterial plasma membrane, coarse-grained modeling, bilayer parameters, cardiolipin, phosphatidylglycerol, molecular dynamics, molecular interactions

## Abstract

In this article, we used molecular dynamics (MD), one of the most common methods for simulations of membranes, to study the interaction of fluorescent membranotropic biological probe 10-*N*-nonyl acridine orange (NAO) with the bilayer, mimicking a plasma membrane of Gram-negative bacteria. Fluorescent probes serve as an effective tool to study the localization of different components in biological membranes. Revealing the molecular details of their interaction with membrane phospholipids is important both for the interpretation of experimental results and future design of lipid-specific stains. By means of coarse-grained (CG) MD, we studied the interactions of NAO with a model membrane, imitating the plasma membrane of Gram-negative bacteria. In our simulations, we detected different NAO forms: monomers, dimers, and stacks. NAO dimers had the central cardiolipin (CL) molecule in a sandwich-like structure. The stacks were formed by NAO molecules interlayered with anionic lipids, predominantly CL. Use of the CG approach allowed to confirm the ability of NAO to bind to both major negatively charged phospholipids, phosphatidylglycerol (PG) and CL, and to shed light on the exact structure of previously proposed NAO–lipid complexes. Thus, CG modeling can be useful for the development of new effective and highly specific molecular probes.

## 1. Introduction

Today, molecular dynamics (MD) is one of the most common methods for simulations of membranes. MD simulations allow to reveal the effects of lipid composition on physicochemical properties of lipid bilayers which mediate membrane interactions with the surrounding. Using the MARTINI coarse-grained (CG) approach dealing with simplified molecular models with reduced number of particles makes it possible to study interactions of small molecules with biological membranes on a time scale of tens of microseconds.

The plasma (or cytoplasmic) membrane, based on a bilayer of phospholipids, is an integral structure of the cells of both Gram-positive and Gram-negative bacteria. In the latter, the plasma membrane is also called the inner membrane, since the cell wall of these bacteria includes an additional outer asymmetric membrane, the inner leaflet of which is built with phospholipids, and the outer leaflet is mainly formed by lipopolysaccharides [1].

Bacterial phospholipids are mainly represented by glycerophospholipids, the molecules of which contain two fatty acid residues, a glycerol moiety, a phosphate group, and a variable head group [2]. The ratio of individual lipids in the plasma membranes of different types of bacteria varies greatly, in particular, lipids can differ in the length and number of acyl chains, bond geometry, and polarity. These variations provide a difference in the biophysical parameters of bacterial membranes, such as fluidity and permeability [3]. In bacterial plasma membranes, the most common glycerophospholipids are zwitterionic phosphatidylethanolamine (PE), which predominates in most Gram-negative bacteria, as well as anionic phosphatidylglycerol (PG) and cardiolipin (CL) [2,4,5,6]. A typical phospholipid composition of Gram-negative *Escherichia coli* from exponential growth phase cultures is described as 75% PE, 20% PG, and 5% CL [7].

Among bacterial phospholipids, CL, which is a derivative of tetraacylated diphosphatidylglycerol with four unsaturated acyl chains and a polar head bearing two negatively charged phosphate groups, is of the greatest interest. Depending on the length and degree of unsaturation of fatty acyl chains, the CL family provides a high potential for adaptation reactions and responses to stress [8]. Depending on the growth phase and cultivation conditions, the relative content of CL among *E. coli* phospholipids can vary within 5–15%, increasing sharply upon transition to the stationary phase of culture growth [9]. In *E. coli*, the products of three genes (*clsA*, *clsB*, and *clsC*) are involved in the CL synthesis [10]. Various abiotic stresses induce transcription of the *clsA* gene, the product of which is the major cardiolipin synthase. Increased osmolality causes excessive accumulation of CL [11], which colocalizes the ProP osmosensory transporter at the poles and near the septa of dividing *E. coli* cells [11,12].

Elevated levels of cardiolipin in the bacterial membrane can mediate resistance to certain antibiotics, such as the lipopeptide daptomycin, by inhibiting octameric pore formation and membrane permeabilization induced by this antibiotic [13]. On the other hand, CL, through electrostatic interactions, can serve as a target for cationic antimicrobials. Thus, the bactericidal activity of the amino-alcohol sphingosine, which is present in the epithelial cells of the nose, trachea, and bronchi, promotes permeabilization of the plasma membrane and death of bacterial cells due to the binding to CL and clustering of CL molecules [14].

The organization of bacterial plasma membranes is characterized by a heterogeneous lateral distribution of phospholipids [6,15]. PE and PG tend to localize in the cylindric regions of the membrane of rod-shaped bacteria, but segregate into different domains [16]. CL molecules having a conical shape tend to form domains at the poles and the septa [17,18], regions with the largest negative membrane curvature, ∼2 μm^−1^ and ∼10 μm^−1^, respectively [19]. In the simulated bilayers, CL also strongly favors membrane regions with negative curvature, while PG favors regions with positive curvature [20].

CL domains play an important role in organizing the spatial distribution and functioning of proteins of the bacterial division machinery [18] and the positioning of the site of cell division [21]. Staining of CL in *E. coli* with the probe erylysin A-EGFP inhibited cytokinesis and caused abnormal distribution of the cytoskeletal protein RodZ. It also revealed CL localization in the inner leaflet of the plasma membrane at cell poles and the outer leaflet at division sites [22]. Interestingly, CL is synthesized at the inner (plasma) membrane of Gram-negative bacteria, but it is also localized in the inner leaflet of the outer membrane, where it is transported by the PbgA protein [23].

The fluorophore 10-*N*-nonyl-3,6-bis(dimethylamino)acridine (10-*N*-nonyl acridine orange, NAO) has been widely used as the specific fluorescent CL probe to observe local concentration of CL in bacteria and mitochondria of eukaryotic cells. In NAO, the NH group in position 10 of acridine orange is replaced by the *N*-nonyl group resulting in the insertion into the lipid bilayer [17]. Due to the positive charge on the acridine ring NAO has the ability to bind electrostatically to the phosphate groups of anionic phospholipids. Even more significant may be a hydrophobic interaction between the nonyl chain of NAO and fatty acid chains of phospholipids [24]. However, only the interaction of NAO with CL, which carry two phosphate groups, was believed to form a complex of CL with two closely located NAO molecules. Such complexes can be detected by the unique red Stokes shift of fluorescence maximum from a green (524 nm) to red (640 nm) spectral region [25]. NAO was also expected to aggregate at hydrophobic domains exposed on the bilayer surface by CL [26]. Later, it was shown that the spectral properties of NAO change in a similar way when bound to other anionic phospholipids [25]. In the absence of CL, in bacteria lacking CL synthases, NAO can also indicate PG localization in the sites typical for CL [27].

Another fluorescent sensor developed for CL quantification was a positively charged 1,1,2,2-tetrakis [4-(2-trimethylammonioethoxy)phenyl]ethene tetrabromide (TTAPE-Me) with aggregation-induced emission [28]. However, it turned out that TTAPE-Me is also not specific for CL and detects PGs with the same efficiency [29].

Thus, although fluorescent probes are of interest for determining the localization of individual phospholipids in biological membranes, their sensitivity and selectivity are far from satisfactory. Understanding the detailed molecular mechanism of dye interactions with membrane phospholipids is important both for interpreting experimental results and the design of more lipid-specific fluorescent probes. Studying the interaction of various dyes with microbial cell wall components at the molecular level is a complex task, which, nevertheless, can be solved using computer simulation, which makes it possible to characterize the details of intermolecular interactions and achieve high efficiency in the study of biological processes.

In this work, we developed a CG model of NAO and used MD to study its interaction with phospholipids in a model CL-containing bacterial membrane. The use of CG modeling made it possible to characterize in the microsecond range the process of NAO binding to a bilayer that mimics the plasma membrane of Gram-negative bacteria. Herein, for the first time, in the computational simulation, we were able to detect the formation of NAO–lipid complexes, which should undoubtedly affect the spectral characteristics of the fluorescent probe.

## 2. Results

### 2.1. Electrostatic Charactersitcs of Coarse-Grained Model Components

**Figure 1:**
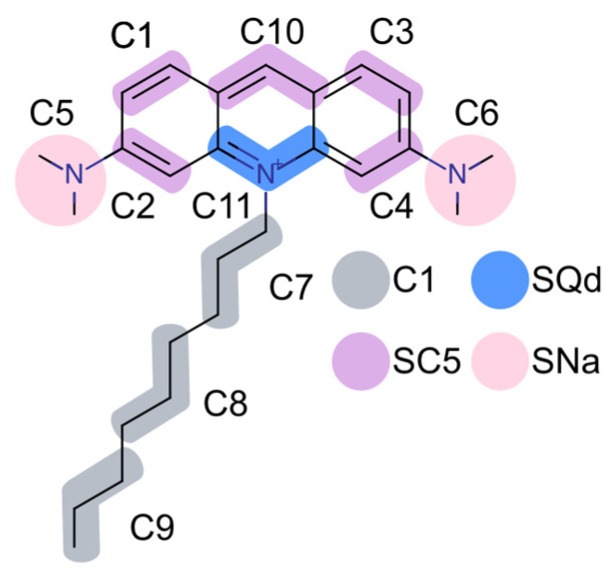
Structural formula of the NAO molecule with the AA to CG mapping according to the MARTINI force field. Bead types are differentiated by color. The bead names are labeled according to CG topology.

**Figure 2:**
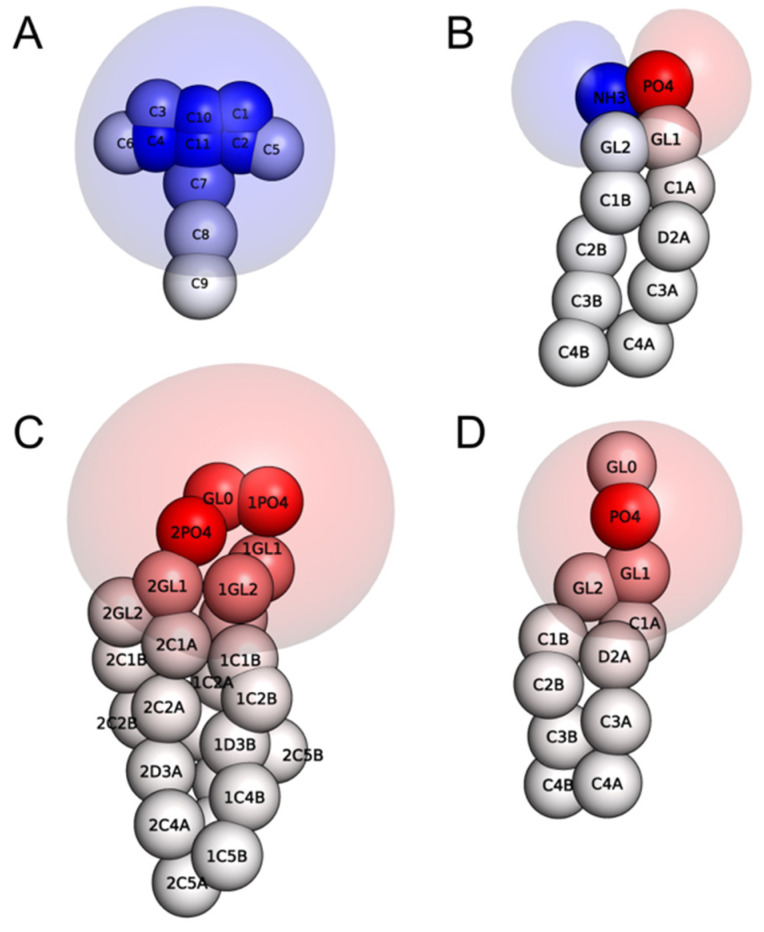
CG spatial structures of NAO and phospholipid components of the model membrane and their equipotential electrostatic surfaces +7 mV (blue), –7 mV (red): NAO (**A**), PE (**B**), CL (**C**), PG (**D**). Colors of CG beads correspond to molecular surface electrostatic potential.

The CG model of the membrane probe NAO was developed in the MARTINI force field, and the chemical structure of NAO with the all-atom (AA) to CG mapping is depicted in Figure 1. The positive equipotential surface of the NAO CG model with one positively charged C11 bead is shown in Figure 2A. The procedure of CG model development is described in the Material and Methods section.

In our MD simulations, the CG model of the bacterial plasma membrane was composed of 384 neutrally charged PE lipids, 48 PG, and 48 CL anionic lipids. Equipotential surfaces of phospholipids (Figure 2B–D) demonstrate the zwitterionic nature of PE, while PG and CL lipids in the MARTINI force field generate a strong negative electric field.

**Figure 3:**
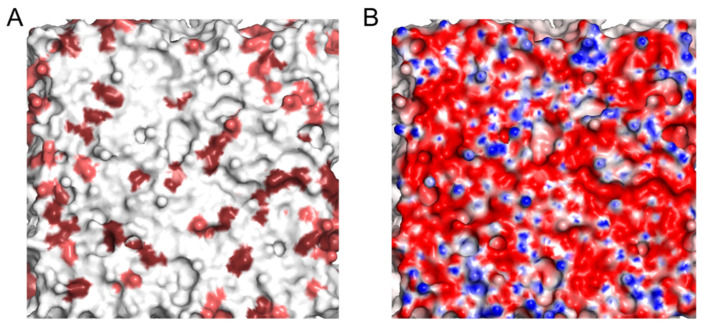
Equilibrated model membrane (**A**) and its surface electrostatic potential (**B**). The molecular surface of bilayer on the panel A is colored according to lipid type (PE—in white, PG—in salmon, CL—in dark ruby); the molecular surface of bilayer on the panel B is colored according to surface electrostatic potential from −100 mV (red) to +100 mV (blue).

The model membrane, which includes anionic PG and CL lipids, has a strong negative charge of 144 elementary charges. A predominantly negative electrostatic potential is generated on its surface, with small positive regions arising from the polar heads of the PE, and few hydrophobic regions, which are probably formed due to the exposure of lipid acyl chains (Figure 3).

### 2.2. Insertion of NAO Molecules into the Model Bilayer

**Figure 4:**
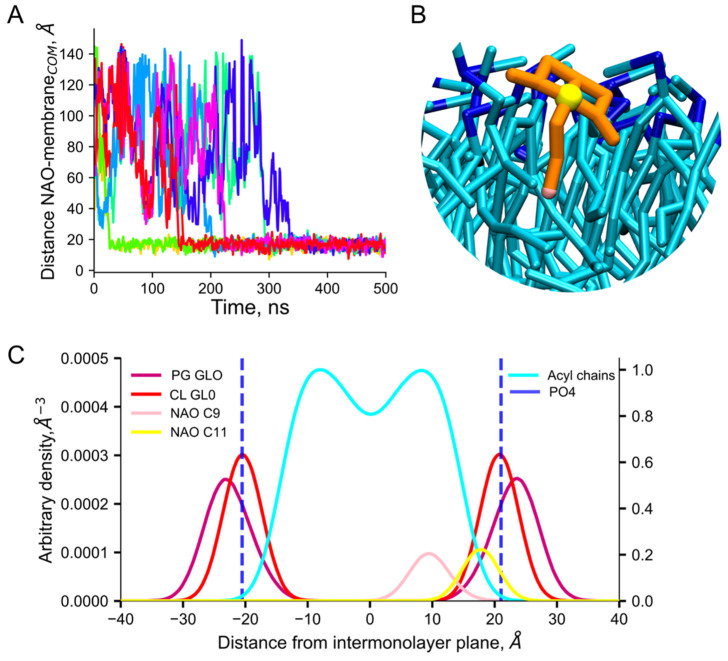
Insertion of NAO into the model bilayer. Distance between the center of mass of modeled bilayer and NAO shown in different colors for individual NAO molecules (**A**). Representative conformation of inserted NAO molecule (charged bead of NAO is shown as yellow sphere; terminal bead of NAO is pink; other NAO beads are shown as orange sticks; the acyl chains of lipids are shown as cyan sticks; lipid phosphates are shown as blue sticks) (**B**). The density profiles of key chemical moieties of NAO and lipids along the membrane normal. Blue dashed lines correspond to the average position of lipid phosphates (**C**).

To study the interaction with the model plasma bacterial membrane, NAO was added in the box with the equilibrated model bilayer at the ratio of 1:60 or 1:6 NAO:lipid. Each MD simulation was carried out for 7 μs. In less than half a microsecond, all NAO molecules inserted into the lipid bilayer (Figure 4A). The NAO hydrophobic acyl tail penetrated into the region of the lipid fatty acid chains. The terminal C9 bead of the NAO model molecules located at a distance of 1 nm from the bilayer center, while the positively charged C11 bead colocalized with the lipid phosphate groups (Figure 4B,C).

**Figure 5:**
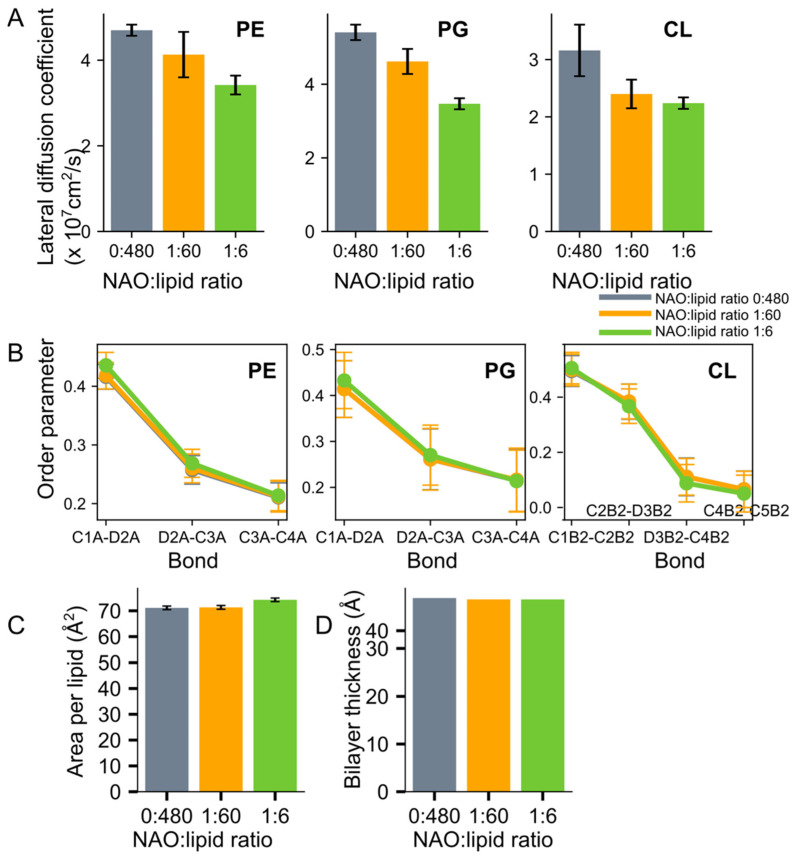
Lateral diffusion coefficients of lipids (**A**), order parameters of lipid acyl chains (**B**), area per lipid (**C**), and average bilayer thickness (**D**) of model membrane at different NAO concentrations.

As a result of NAO insertion into the lipid bilayer at a low concentration (NAO:lipid 1:60), the lateral diffusion coefficient of neutral lipid PE decreased to a lesser extent, while the greatest change was observed for CL (Figure 5A). At the increased NAO concentration (NAO:lipid 1:6), the lateral diffusion coefficients decreased further only for PE and PG. As a result, the decrease of lateral diffusion coefficients of all membrane lipids was within 30 percent. The NAO insertion into the model bilayer did not influence the order parameters of lipid acyl chains (Figure 5B) and had little effect on the area per lipid and the average bilayer thickness (Figure 5C,D).

### 2.3. Interaction of NAO with Lipids Lead to the Formation of Complexes with Different Composition

**Figure 6:**
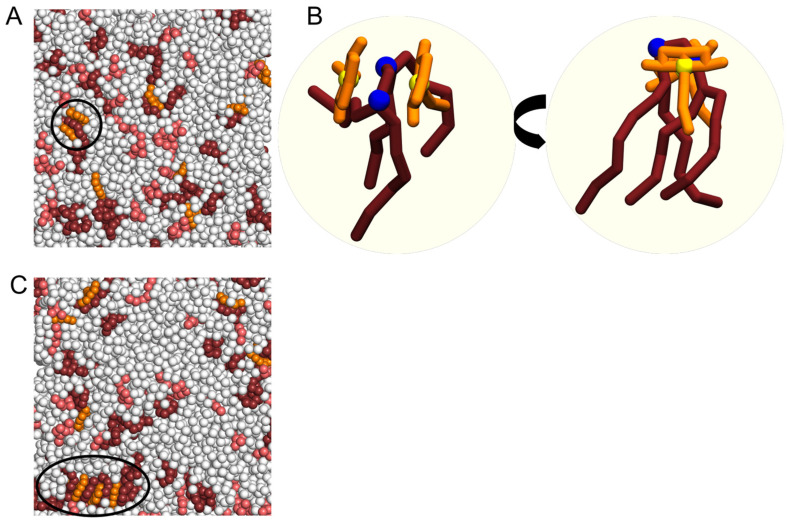
Formation of complexes of NAO with anionic lipids at 1:60 NAO:lipid ratio. PE is colored in white, PG—in salmon, CL—in dark ruby, NAO—in orange (**A**). Enlarged view of complex consisting of two NAO molecules and one CL molecule (**B**). Formation of heterogeneous stack composed of NAO and CL molecules (**C**). Black circles highlight sandwich-like complexes of NAO with anionic lipids.

After NAO insertion into the bilayer (NAO:lipid 1:60), NAO molecules exist as monomers or form complexes with anionic PG and CL lipids. During MD simulation, we observed formation of complexes consisting of two NAO molecules and one CL molecule (Figure 6A,B). In some cases, heterogeneous stacks composed of a few NAO and CL molecules took place (Figure 6C).

**Figure 7:**
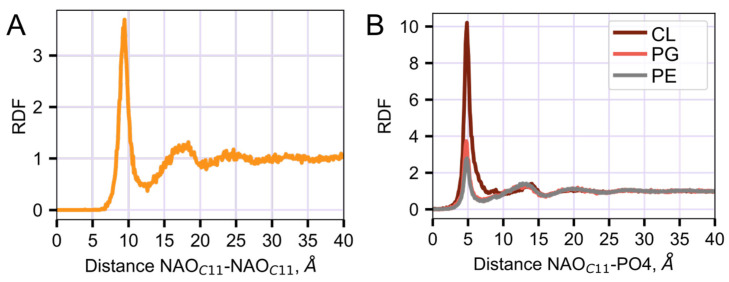
2D RDFs of distances between the NAO C11-charged beads (**A**) and NAO C11-charged beads and phosphates of different lipids (**B**) calculated for the ratio 1:6 NAO:lipid.

The existence of NAO in complexes of two NAO molecules and one CL, or in stacks, in which NAO molecules are layered with CL, was confirmed by radial distribution function (RDF) analysis. On RDF, the peak at about 9.4 Å (Figure 7A) corresponds to the distance between the NAO C11-charged beads colocalized with the CL molecule in the sandwich-like structure. RDF analysis shows that NAO binds preferentially to CL. This is evident from the larger CL peak magnitude (at 5 Å), which corresponds to a distance between the C11 NAO bead and phosphate groups of different lipids (Figure 7B).

**Figure 8:**
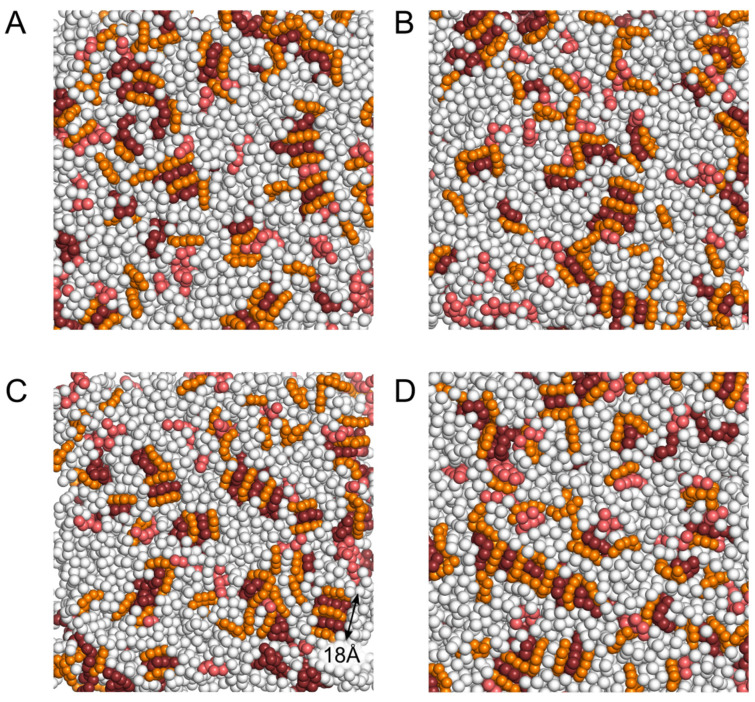
Snapshots demonstrating formation of heterogeneous stacks composed of NAO and anionic lipids when NAO is added to the membrane in the ratio of 1:6 NAO:lipid. PE is colored in white, PG—in salmon, CL—in dark ruby, NAO—in orange. Panels (**A**–**D**) correspond to different MD snapshots. Black double end arrow indicates the size of 3NAO:2CL heterogeneous stack.

The probability of stack formation between NAO and anionic lipids increased in the computational experiment with a higher concentration of NAO (NAO:lipid 1:6). We observed heterogeneous stacks composed of a few NAO and anionic lipids, in which NAO interacted mainly with CL, but PG could also be present in such stacks (Figure 8). The simplest stacks with a size of about 9.4 Å contained two NAO molecules and one CL molecule. The size of more complex stacks formed by three NAO and two CL molecules was of about 18 Å (Figure 8C). We have also observed more complex stacks that result from combining such aggregates. The stacks had a sandwich-like structure in which NAO and CL molecules were interlayered.

## 3. Discussion

Selective fluorescent probes, especially those with red and green light, are widely used to visualize biomolecules, organelles, whole cells, and tissues. Among these dyes, one of the most studied is acridine orange (AO). This basic acridine dye intercalates into double-stranded DNA in a monomeric form with green fluorescence, but forms red fluorescent aggregates when it is coordinated with single-stranded nucleic acids [30,31]. In aqueous solutions at concentrations below 10 µM, AO is present predominantly in the monomeric form with an absorption maximum at 490 nm and the shoulder at 468–470 nm. The absorption intensity at 468 nm increases at higher AO concentrations compared to that at 490 nm due to the formation of AO dimers and high-order aggregates. The fluorescence maxima of AO monomers and aggregates are at 529 and 634 nm, respectively [32,33].

*N*-alkyl acridine orange derivatives are formed by replacing the NH group in position 10 of acridine orange by the *N*-alkyl group. This leads to the loss of the ability to interact with nucleic acids and the appearance of the ability to interact with negatively charged phospholipids [17]. The best-known compound of this type used for CL imaging is NAO, but other *N*-alkyl derivatives have been synthesized and tested with some success for this purpose [34,35].

According to [17], during the incubation of *E. coli* bacteria with NAO, a fluorescent signal was observed in the green and red regions of the spectrum, which may indicate both the binding of NAO in the monomeric form to CL as well as the binding to PG. The high ability of NAO to bind to CL with a stoichiometry of 2 mol NAO/mol CL has been explained [24,26,36] by electrostatic interactions between the quaternary ammonium of NAO and two ionized phosphate groups of CL, as well as hydrophobic interactions between neighboring chromophores and NAO alkyl chain with fatty acids of CL.

One of the most popular methods for studying biological systems with molecular modeling is MD. Using MD, it is possible to obtain a set of frames corresponding to a specific modeling step and follow the changes in the positions of particles in the process of system evolution. In our MD simulations, we observed the process of NAO insertion in the bilayer as the following: electrostatic attraction of positively charged acridine moiety of NAO to bilayer with negative surface potential, rotation of NAO molecule so that its hydrophobic tail becomes parallel to the plane of the model membrane, insertion of the tail between the acyl chains of lipids. Being inserted in the bilayer with its *N*-alkyl chain (Figure 4B), the positively charged NAO head group (bead C11 in Figure 1) is able to form electrostatic encounter complexes with negatively charged phosphate groups of lipids. In such complexes, the distance between NAO C11 and phosphate is about of 5 Å (Figure 7B). The probability to find NAO C11 beads at the distance of 5 Å from the phosphate groups depends on the lipid type; it decreases in the row CL, PG, PE (Figure 7B), which corresponds to the electrostatic potential properties of these phospholipids (Figure 2).

In addition to complexes of NAO:CL and NAO:PG in a stoichiometric ratio of 1:1, MD shows the formation of 2NAO:1CL complexes with the central CL molecule in a sandwich-like structure (Figure 6A,B), according to the proposed structural mode of interaction between AO-type dyes and CL [24]. Even at a low concentration of NAO (NAO:lipid 1:60), one could observe the formation of higher-order complexes in the form of stacks, in which NAO molecules are interlayered by CL molecules (Figure 6C). Obviously, the formation of complexes 2NAO:1CL and higher-order stacks of NAO and CL is reflected in the 9.4 Å RDF peak for the distance between C11 beads of two NAO molecules (Figure 7A). Both lower-order complexes and stacks are dynamic structures that quickly transform and break down.

With an increase in the concentration of NAO by an order of magnitude (NAO:lipids 1:6), the stacks become common, complex, and highly heterogeneous in composition (Figure 8). Despite the fact that the vast majority of NAO molecules are found precisely in such complex stacks, predominantly with CL, NAO can be found both in complexes with PG and in the form of monomers (Figure 8). The results of our simulations confirm the ability of NAO to bind to both major negatively charged lipids, CL and PG, which was previously observed in the experiments [17,25,27,37]. Nevertheless, it should be noted that, according to our data, the probability to find NAO in a complex with CL is much higher than that with PG (Figure 7B). Thus, with the help of CG modeling and MD, we have shown the existence of all forms of NAO in complexes with CL and PG predicted from the results of experimental studies. Such a complex system of NAO interactions with various anionic lipids should undoubtedly affect the spectral properties of this biological stain.

The lipid content of our model bilayer reflects the real ratio of lipids at the poles of Gram-negative rod-shaped *E. coli* cells with almost the same relative abundance of CL and PG [38]. When choosing NAO concentrations for computational experiments, it was taken into account that NAO is used for staining bacterial cells at concentrations of about 2 μM [25], and that the phospholipid concentration in biological samples is no more than 8 μM [24]. When NAO interacts with CL and PG, its red fluorescence is at a maximum and the green fluorescence is at a minimum at a stoichiometry of 2:1 for NAO-CL and 1:1 for NAO-PG [25]. In our simulations, the maximal ratio was 1:6 in relation of NAO to the total number of lipid molecules, and NAO was approximately equal to the anionic lipid content (CL + PG). We did not further increase the amount of NAO, since, at such concentrations, there was already a noticeable effect of NAO on the characteristics of the model bilayer. Using plausible model systems mimicking bacterial membranes and their staining with NAO, allowed us to obtain characteristics of intermolecular interactions that are in good agreement with the experimental data.

Finally, in this study, we characterized the molecular interactions between the organic cation NAO and membrane composed of zwitterionic and anionic phospholipids, and described different types of NAO complexes with individual lipids. Our study shows that, despite the simplification used in the CG approach, the model membrane systems obtained by this method reflect the real processes of interaction between membranotropic compounds and bilayers and, thus, can be extremely useful for the development of membrane probes, improving their sensitivity and lipid specificity.

## 4. Materials and Methods

The CG approach deals with simplified molecular models as compared to the AA modeling. The most popular MARTINI force field [39] is based on the assumption that groups of several heavy atoms of certain chemical groups can be combined to represent one CG particle. This force field appeared to be an effective approach in studying various biomolecular systems. In this study, the MARTINI force field was used to develop the CG NAO model. The CG beads were mapped onto the AA chemical structure, and their types were chosen by analogy with the already parametrized methylene blue molecule [40] and lipid acyl chains [39]. The mapping scheme is shown in Figure 1. The CG model of NAO consists of 11 beads for which 12 bonds, 19 bond angles, and 4 dihedral angles have been described. Only the central C11 bead corresponding to acyl tail attachment site was modeled charged (+1). The model bonded term parameters were iteratively optimized based on the all-atom (AA) MD simulation according to the procedure we used previously [41]. Since the acridine ring structure is very rigid and highly packed, it experiences a heightened strain. To reduce this strain, we use the nonbonded exclusions between all of the CG beads forming the acridine orange ring. We also replaced almost all bonded terms with the constraints to achieve convergence between AA- and CG-bonded terms distribution.

The AA topology of NAO was created using the ATB web-service [42] and GROMOS9654a7 [43]. A single NAO molecule was added into the box with the dimensions of 5.55 nm × 4.92 nm × 4.35 nm and an appropriate number of SPC water molecules and Na+/Cl− ions, resulting in the ionic strength of 150 mM and zero total charge. The auxiliary AA simulation was run in the NPT ensemble for 400 ns (T = 303.15 K maintained by the V-rescale algorithm, P = 1 bar controlled by the Parrinello-Rahman barostat; the integration time step was set to 2 fs). Figures S1–S3 demonstrate satisfactory agreement between AA and CG models. Topology and structure files are available in the Supporting Information (Text S1).

In this study, we used the CG model of the plasma membrane of bacteria, consisting of PE, PG, and CL at a ratio of 8:1:1. The ratio of lipids in the model bilayer was close to the lipid composition of bacterial plasma membranes in the poles of the Gram-negative rod-shaped *E. coli* [25,38]. The model bilayer was assembled in a CHARMM-GUI Martini Maker [44]. The electrostatic potential fields of NAO, phospholipids, and equilibrated bilayer were calculated using Poisson–Boltzmann formalism in ProKSim software at ionic strength 100 mM [45]. On the basis of these calculations, we created the equipotential surfaces of individual molecules of +/−7 mV. The CG spheres of individual molecules and the surface of the equilibrated bilayer were colored according to the surface electrostatic potential from −100 mV (red) to +100 mV.

NAO molecules were added to the model equilibrated membrane at two different concentrations, 1:60 and 1:6 NAO:lipid ratio. Each production MD simulation was prefaced by the steepest descent minimization. The details of the production CG simulations are provided in Table S1. The simulation parameters were chosen according to recommendations [46]. The MD simulations were run in the NPT ensemble using the V-rescale thermostat (T = 320 K, τ_t_ = 1.0 ps) and the Parrinello-Rahman barostat (time constant = 12.0 ps, compressibility = 3 × 10^−4^ bar^−1^, applied semi-isotropically). CG simulations were performed with the polarizable water (PW) model (ε_r_ = 2.5) with the addition of the appropriate number of Na+/Cl− ions to achieve ionic strength of 150 mM [47]. The integration time step was 20 fs. MD simulations were performed using Gromacs 2019.4 [48]. The parameters of the modeled membrane (area per lipid, average membrane thickness, lipid diffusion coefficients, order parameter of lipid acyl chains, and RDF) in the absence and presence of NAO were calculated as previously described [49].

## Data Availability

Not applicable.

## Supplementary Materials

The following supporting information can be downloaded at: https://www.mdpi.com/article/10.3390/molecules28041929/s1, Figure S1: Distribution of the bond terms from AA (blue) and CG (red) simulations for NAO. The numbers of CG bead IDs for which the bond is described are given on each panel according to the attached topology; Figure S2: Distribution of the angle terms from AA (blue) and CG (red) simulations for NAO. The numbers of CG bead IDs for which the angle is described are given on each panel according to the attached topology; Figure S3: Distribution of the dihedral terms from AA (blue) and CG (red) simulations for NAO. The numbers of CG bead IDs for which the dihedral is described are given on each panel according to the attached topology. Table S1: Molecular composition of simulated systems.; Text S1: CG structure (PDB format) and topology (Gromacs ITP files) of NAO.
