# Supplementary material for: Insights into the Formation of Intermolecular Complexes of Fluorescent Probe 10-N-Nonyl Acridine Orange with Cardiolipin and Phosphatidylglycerol in Bacterial Plasma Membrane by Molecular Modeling
Source: Molecules. 2023 Feb 17;28(4):1929. doi: 10.3390/molecules28041929 (PMC9961436; doi:10.3390/molecules28041929)
Supplement: Supplementary file 1 [file molecules-28-01929-s001.zip › molecules-2228234-supplementary.pdf]

Insights into the Formation of Intermolecular Complexes of Fluorescent Probe 10-N-nonyl Acridine Orange with Cardiolipin and Phosphatidylglycerol in Bacterial Plasma Membrane by Molecular Modeling

Ekaterina Kholina, Ilya Kovalenko, Andrew Rubin and Marina Strakhovskaya

Faculty of Biology, Lomonosov Moscow State University, 119234 Moscow, Russia; tenarra1@gmail.com (E.K.); styx@biophys.msu.ru (S.K.); ikovalenko78@gmail.com (I.K.); rubin@biophys.msu.ru (A.R.); maristra@yandex.ru (M.S.)

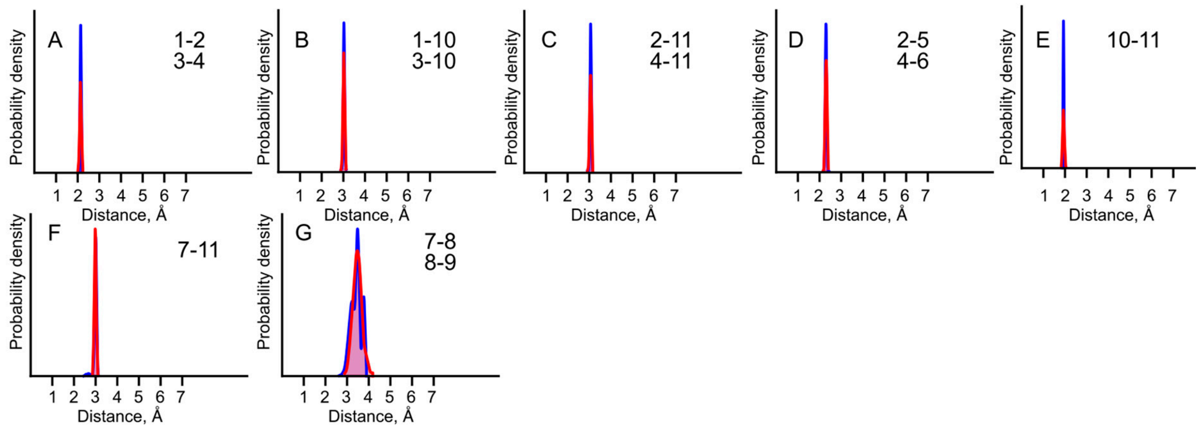

Figure S1. Distribution of the bond terms from AA (blue) and CG (red) simulations for NAO. The numbers of CG bead IDs for which the bond is described are given on each panel according to the attached topology.

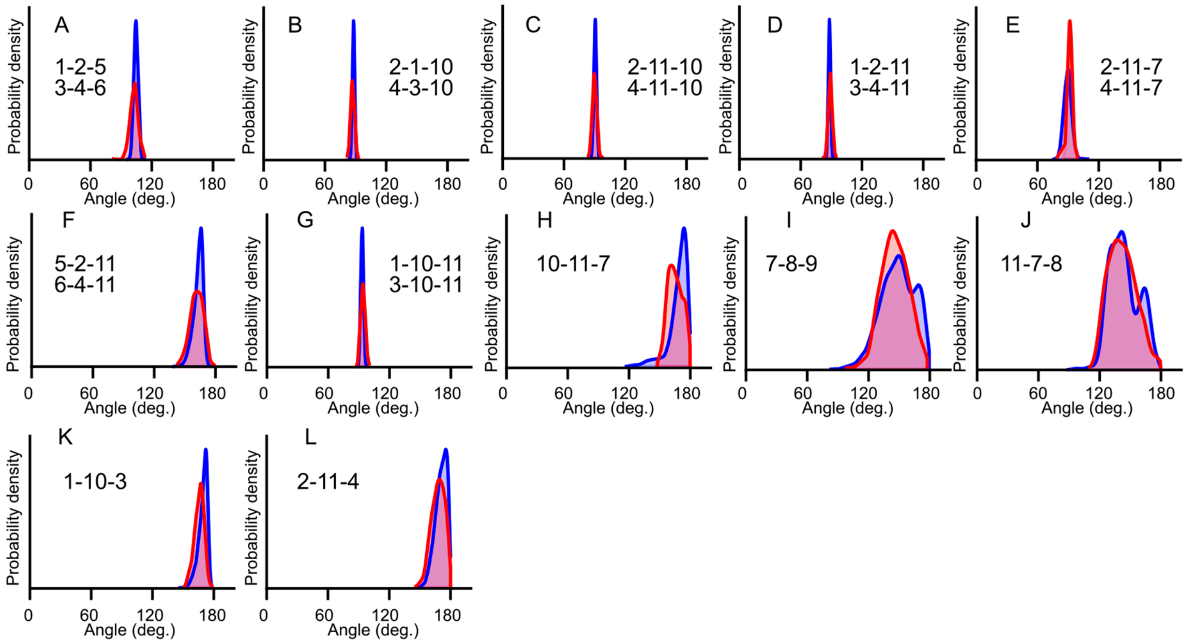

Figure S2. Distribution of the angle terms from AA (blue) and CG (red) simulations for

NAO. The numbers of CG bead IDs for which the angle is described are given on each panel according to the attached topology.

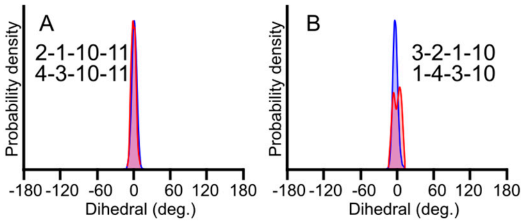

Figure S3. Distribution of the dihedral terms from AA (blue) and CG (red) simulations for NAO. The numbers of CG bead IDs for which the dihedral is described are given on each panel according to the attached topology.

Table S1. Molecular composition of simulated systems.

Number of lipids and NAO molecules	Number of PW molecules	Number of ions (Na^+ / Cl–)	Box dimensions (x,y,z), nm
POPE:POPG:CDL2 384:48:48	14044	228 / 156	13.14, 13.14, 14.32
POPE:POPG:CDL2: NAO 384:48:48:8	27112	510 / 374	13.15, 13.15, 22.70
POPE:POPG:CDL2: NAO 384:48:48:80	33835	532 / 468	13.38, 13.38, 26.68

Text S1. CG structure (PDB format) and topology (Gromacs ITP files) of NAO.

```
NAO.pdb
ATOM    1  C1  NAO    1     22.500  47.000  33.050  1.00  0.00
ATOM    2  C2  NAO    1     21.760  45.200  33.930  1.00  0.00
ATOM    3  C3  NAO    1     16.930  49.140  32.540  1.00  0.00
ATOM    4  C4  NAO    1     16.110  47.490  33.610  1.00  0.00
ATOM    5  C5  NAO    1     23.750  44.160  34.460  1.00  0.00
ATOM    6  C6  NAO    1     13.880  47.840  33.130  1.00  0.00
ATOM    7  C7  NAO    1     17.930  43.680  34.950  1.00  0.00
ATOM    8  C8  NAO    1     17.170  41.690  38.240  1.00  0.00
```

```
ATOM      9  C9  NAO     1      16.150  39.550  40.850  1.00  0.00
ATOM     10  C10 NAO     1      19.620  47.900  33.150  1.00  0.00
ATOM     11  C11 NAO     1      18.910  46.330  33.990  1.00  0.00
END
```

NAO.itp

```
;CG topology of NAO
[ moleculetype ]
; molname       nrexcl
NAO             1

[ atoms ]
; id type resnr residue   atom  cgnr    charge
1     SC5     1     NAO     C1  1      0.00000
2     SC5     1     NAO     C2  2      0.00000
3     SC5     1     NAO     C3  3      0.00000
4     SC5     1     NAO     C4  4      0.00000
5     SNa     1     NAO     C5  5      0.00000
6     SNa     1     NAO     C6  6      0.00000
7      C1     1     NAO     C7  7      0.00000
8      C1     1     NAO     C8  8      0.00000
9      C1     1     NAO     C9  9      0.00000
10    SC5     1     NAO     C10  10     0.00000
11    SQd     1     NAO     C11  11     1.00000


[ bonds ]
;   i    j   funct   length   force.c.
    7    8      1    0.361  6382.88
    8    9      1    0.361  6382.88

 [ constraints ]
  1   2 1 0.213
  3   4 1 0.213
  1  10 1 0.302
  3  10 1 0.302
  2  11 1 0.306
  4  11 1 0.306
  2   5 1 0.230
  4   6 1 0.230
 10  11 1 0.193
  7  11 1 0.298

[ angles ]
;   i    j    k   funct     angle    force.c.
    1    2    5      2    103.66     686.98
    3    4    6      2    103.66     686.98
    2    1   10      2     89.08     638.35
    4    3   10      2     89.08     638.35
```

2	11	10	2	91.90	1272.37
4	11	10	2	91.90	1272.37
1	2	11	2	89.49	745.98
3	4	11	2	89.49	745.98
2	11	7	2	79.42	216.70
4	11	7	2	79.42	216.70
5	2	11	2	171.30	466.47
6	4	11	2	171.30	466.47
1	10	11	2	93.46	598.57
3	10	11	2	93.46	598.57
10	11	7	2	173.63	1133.32
7	8	9	2	155.14	100.41
11	7	8	2	152.45	117.55
1	10	3	2	167.11	1339.00
2	11	4	2	162.75	40.93

[dihedrals]

; i	j	k	l	funct	dihedral	force.c.	mult.
2	1	10	11	2	-0.31	685.88	
4	3	10	11	2	-0.31	685.88	
3	2	1	10	2	-2.84	192.15	
1	4	3	10	2	-2.84	192.15	

[exclusions]

; i	j						
1	2	3	4	5	6	10	11
2	3	4	5	6	10	11	
3	4	5	6	10	11		
4	5	6	10	11			
5	6	10	11				
6	10	11					
10	11						
